# Seasonal Occurrence and Carbapenem Susceptibility of Bovine *Acinetobacter baumannii* in Germany

**DOI:** 10.3389/fmicb.2019.00272

**Published:** 2019-02-22

**Authors:** Peter Klotz, Paul G. Higgins, Andreas R. Schaubmar, Klaus Failing, Ursula Leidner, Harald Seifert, Sandra Scheufen, Torsten Semmler, Christa Ewers

**Affiliations:** ^1^Institute of Hygiene and Infectious Diseases of Animals, Justus Liebig University Giessen, Giessen, Germany; ^2^Institute for Medical Microbiology, Immunology and Hygiene, University of Cologne, Cologne, Germany; ^3^German Center for Infection Research (DZIF), Partner Site Bonn-Cologne, Cologne, Germany; ^4^Unit for Biomathematics and Data Processing, Faculty of Veterinary Medicine, Justus Liebig University Giessen, Giessen, Germany; ^5^Institute for Hygiene and Microbiology, University of Würzburg, Würzburg, Germany; ^6^NG1 Microbial Genomics, Robert Koch Institute, Berlin, Germany

**Keywords:** ESKAPE, *Acinetobacter baumannii*, antimicrobial susceptibility, MLST, cattle, epidemiology

## Abstract

*Acinetobacter baumannii* is one of the leading causes of nosocomial infections in humans. To investigate its prevalence, distribution of sequence types (STs), and antimicrobial resistance in cattle, we sampled 422 cattle, including 280 dairy cows, 59 beef cattle, and 83 calves over a 14-month period. Metadata, such as the previous use of antimicrobial agents and feeding, were collected to identify putative determining factors. Bacterial isolates were identified via MALDI-TOF/MS and PCR, antimicrobial susceptibility was evaluated via VITEK2 and antibiotic gradient tests, resistance genes were identified by PCR. Overall, 15.6% of the cattle harbored *A. baumannii*, predominantly in the nose (60.3% of the *A. baumannii* isolates). It was more frequent in dairy cows (21.1%) than in beef cattle (6.8%) and calves (2.4%). A seasonal occurrence was shown with a peak between May and August. The rate of occurrence of *A. baumannii* was correlated with a history of use of 3rd generation cephalosporins in the last 6 months prior to sampling Multilocus sequence typing (Pasteur scheme) revealed 83 STs among 126 unique isolates. Nine of the bovine STs have previously been implicated in human infections. Besides known intrinsic resistance of the species, the isolates did not show additional resistance to the antimicrobial substances tested, including carbapenems. Our data suggest that cattle are not a reservoir for nosocomial *A. baumannii* but carry a highly diverse population of this species. Nevertheless, some STs seem to be able to colonize both cattle and humans.

## Introduction

*Acinetobacter* is widespread in the microbiota of animals, plants and the environment (Doughari et al., 2011). Several species are able to cause opportunistic, mainly hospital-acquired infections. In contrast to many other *Acinetobacter* species, *Acinetobacter baumannii* is mainly associated with clinical environments and hospital outbreaks (Towner, 2009). Nevertheless, the bacterium is sporadically found in samples of cattle (Hamouda et al., 2008, 2011; Nam et al., 2009, 2010; Al Bayssari et al., 2015; Rafei et al., 2015; Fernando et al., 2016) and even carbapenem-resistant strains have been reported (Al Bayssari et al., 2015; Pailhoriès et al., 2016; Webb et al., 2016). The increase of carbapenem-resistant *A. baumannii* isolates implicated in human infections causes a serious limitation of treatment options and has been associated with high mortality rates (Falagas and Rafailidis, 2007; Perez et al., 2007). In *Acinetobacter* spp., carbapenem-resistance is mostly mediated by acquired class D beta-lactamases, so called oxacillinases (mainly OXA-23_,_ OXA-40, and OXA-58). A further mechanism is the insertion of a genetic element (e.g., IS*Aba1*) upstream of intrinsic resistance genes, such as the β-lactamase gene *bla*_*OXA*–51–*like*_ which results in its overexpression (Turton et al., 2006). *A. baumannii* strains involved in nosocomial infections frequently belong to certain clonal groups. Eight different so called international clones (ICs) have been identified worldwide, among which the most important groups are IC1 and IC2 corresponding to the multilocus sequence types ST1 and ST2 of the Pasteur scheme (Higgins et al., 2010a). In addition to these major clones important further groups are responsible for mostly regionally distributed outbreaks, e.g., IC7 (corresponding to ST25). Some clonal complexes have not yet been linked to certain ICs although they are also responsible for nosocomial infections and outbreaks, e.g., CC32 which does not belong to IC1-8 (Da Silva et al., 2014; Sahl et al., 2015). *A. baumannii* has also been associated with infections in hospitalized cats, dogs and horses (Endimiani et al., 2011; Zordan, 2011; Smet et al., 2012; Belmonte et al., 2014; Pomba et al., 2014; Ewers et al., 2017) and recent studies have even described the emergence of carbapenem-resistant *A. baumannii* isolates belonging to ST1 and ST2 in companion animals (Pomba et al., 2014; Ewers et al., 2016, 2017). According to the few recent reports, mostly novel STs have been identified among bovine *A. baumannii* isolates, while strains of the dominant clonal lineages were only rarely identified (Lupo et al., 2014; Al Bayssari et al., 2015; Rafei et al., 2015). In order to elucidate the occurrence of *A. baumannii* in livestock, we performed an explorative representative study in the federal state of Hesse, Germany, including the collection of metadata concerning the animals and farms. By that we were able to determine the prevalence of *A. baumannii* in cattle in Hesse and could provide an insight into the phylogenetic diversity of the isolates and the antimicrobial resistance features of bovine *A. baumannii* in German cattle. The analysis of metadata allowed us to identify factors influencing the prevalence of *A. baumannii* and provides important insights for future investigations concerning the prevalence and origin of *A. baumannii*.

## Materials and Methods

### Study Design

We designed our study population based on the total number of 467,787 animals obligatory registered in the national database for cattle ^1^ in Hesse in October 2014. The cattle population in Hesse consists of various breeds, whereof a minority is usually held extensively (Salers, White Park, Scottish Highland, Welsh-Black, Galloway, Belted Galloway, Luing, Small Zebu, White Galloway, Longhorn, Brahman, Heck cattle, Beefalo, Water Buffalo, Bison, European Bison, other crossbreds, and other taurine cattle). Due to a high level of time and effort for sampling these animals, we excluded them from our study. To determine a representative sample size for the Hessian cattle population, the 383,870 animals of usually non-extensively held breeds were then defined as the sampling population. We did not define further exclusion criteria. Taking into account retrospective data from the microbiological diagnostics unit of our institute (P. Klotz, E. Prenger-Berninghoff, S. Scheufen, and C. Ewers, unpublished data), we estimated a response distribution for the occurrence of *A. baumannii* of 1%. Accordingly, a sample size of 380 animals was calculated (confidence level: 95%, margin of error: 1%). Stratification of the sample was done by using the categories “dairy” (female individuals of dairy breeds >7 months), “beef” (male individuals of beef cattle breeds >7 months) and “calf” (male and female individuals <8 months). Furthermore, stratification of the random sample was done according to the number of registered animals in the respective 22 districts (Supplementary Table S1). All animals of the study population were listed by their stratification criteria, and random numbers were assigned to each individual. By sorting the animals according to their random numbers, the animals and their corresponding farms were selected, beginning with the smallest random individual number. Due to matters of time and availability, the individual animals were then conveniently selected by the sampler at the farm. The number of animals on each farm was dependent on the original random list.

### Bacterial Strains, Species Identification

From January 2015 to February 2016 NS and RS from cattle (*n* = 422) as well as FS from the corresponding stables (*n* = 353) were collected in Hesse, Germany (Supplementary Table S4). The FSs were collected at five locations in the stable to increase the chance of finding isolates. The number of 422 animals includes 42 additional samples (28 dairy cows, 9 beef cattle, and 10 calves) to the determined sample size (*n* = 380) due to variable sampling conditions on different farms. The samples were cultured on blood agar (blood agar base by Merck Chemicals, GmbH, Darmstadt, supplemented with 5% sheep blood), Water-blue Metachrome-yellow Lactose Agar (Oxoid, Wesel, Germany), and MacConkey Agar (Oxoid, Wesel, Germany) containing 1 mg/L cefotaxime (Sigma-Aldrich/Merck, Darmstadt, Germany). Colonies morphologically similar to *A. baumannii* were identified using matrix-assisted laser desorption/ionization time-of-flight mass spectrometry (MALDI-TOF/MS; Bruker Daltonics, Bremen, Germany, Database V4.0.0.1). Score values >2.000 were accepted for species identification. The MALDI-TOF/MS results were verified by multiplex PCR targeting different portions of the *gyrB* gene (Higgins et al., 2010b).

### Metadata and Statistical Methods

Metadata concerning the animals and farms were collected and assessed via a questionnaire (Supplementary Table S2). Among other things, questions addressed animal age, sex and breed, farm size, animal feeding, use of sewage sludge as fertilizer, and use of antimicrobials previous to the sampling of animals. For statistical analyses, putative determining factors were identified (Supplementary Table S3). To assess the association of these factors and the prevalence of *A. baumannii* (animals positive either in nasal or RS), a logistic regression model analysis using the generalized linear model (glm) family was performed in two steps. First, due to the high number of variables, the putative determining factors were included separately in the model (single-factor analysis) to evaluate raw associations with the prevalence of *A. baumannii*. Secondly, variables with more than 380 observations (90% of 422 possible observations) and statistical relevant values in the single-factor model (*p* ≤ 0.05) were included in a multiple logistic regression model together. Factors closely associated to the variable category were excluded from the multiple model. As the prevalence of *A. baumannii* was significantly higher in dairy cows, we also performed single-factor and multiple logistic regression analysis exclusively on this group. Criteria for inclusion of variables into the multiple model were a minimum of 252 observations (90% of 280 possible observations) and statistical relevant values (*p* ≤ 0.05) in the single-factor regression analysis. In the same manner we analyzed the occurrence of *A. baumannii* in FSs. Here, variables with more than 318 observations (90% of 353 possible observations) were included to the multiple model. Comparison of the prevalence of *A. baumannii* in different sample locations was conducted via frequency tables and Pearson’s chi-squared test or the fisher’s exact test for count data. The statistical analyses were done by means of the statistical program package R (Free Software Foundation’s GNU project, official homepage^2^).

### Screening for Carbapenem Non-susceptible Strains, Determination of Minimum Inhibitory Concentrations (MICs)

Screening for carbapenem non-susceptible *A. baumannii* was done by streaking the isolates on Mueller-Hinton agar plates (Oxoid, Wesel, Germany) containing meropenem (Sigma-Aldrich/Merck, Darmstadt, Germany) at different concentrations (2 mg/L and 4 mg/L). Minimum inhibitory concentrations (MICs) were determined by using the VITEK2 system and antimicrobial susceptibility testing card AST-GN38 (bioMérieux, Nürtingen, Germany). Imipenem MICs for *A. baumannii* isolates that showed growth on Mueller-Hinton agar containing meropenem were additionally evaluated by using antibiotic gradient agar diffusion method (Liofilchem, Roseto degli Abruzzi, Italy). MICs were interpreted according to CLSI breakpoints defined for human *Acinetobacter* spp. (CLSI, 2014) with exception of nitrofurantoin (breakpoints for Enterobacteriaceae, CLSI), cefpodoxime and ceftiofur (breakpoints for ceftazidime, CLSI), cefpirome (breakpoints for cefepime, CLSI), enrofloxacin and marbofloxacin (breakpoints for ciprofloxacin, CLSI). Intrinsic resistance was assumed according to the definitions described in the EUCAST and CLSI guidelines (CLSI, 2014; EUCAST, 2018).

### Analysis of Antimicrobial Resistance Genes

All isolates were screened for carbapenemase genes *bla*_*OXA*–23,–40,–58_ via PCR (Gröbner et al., 2009). PCRs targeting the IS*Aba1* region upstream of intrinsic *bla*_OXA–51–like_ genes (Turton et al., 2006) and *bla*_KPC_, *bla*_V IM_ (Gröbner et al., 2009), *bla*_OXA–48_ (Poirel et al., 2004), *bla*_NDM_ (Pfeifer et al., 2011), *bla*_IMP_, *bla*_GIM_, *bla*_SIM_, *bla*_SPM_ (Mendes et al., 2007), and *bla*_GES_ (Rieber et al., 2017) were performed on isolates which showed growth on Mueller-Hinton agar containing meropenem, which was used to screen for putative carbapenem-resistant strains.

### Clonal Analysis, Assignment of International Clones and Multilocus Sequence Typing

To identify copy strains among isolates originating from the same farm, the same animal or the same sample location, pulsed-field gel electrophoresis (PFGE) of ApaI-restricted whole genomic DNA was performed (Seifert et al., 2005). International clones I-III were identified via PCR (Turton et al., 2007). Multilocus sequence typing (MLST) was performed according to the Pasteur scheme (Diancourt et al., 2010). A minimum spanning tree based on MLST allele profiles was created with Bionumerics version 6.6 (Applied Maths, Sint-Martens-Latem, Belgium). eBurst v3 was used to assess clonal groups based on allele sequence profiles of 1161 currently available STs^3^ (date of accession: 12-05-2018).

## Results

### Prevalence of *A. baumannii* in 422 Cattle

**FIGURE 1:**
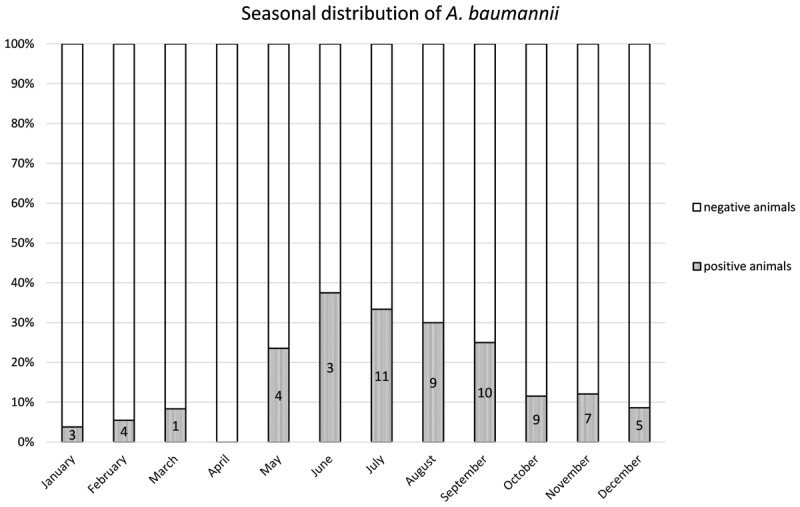
Distribution of the prevalence over the 12 months. Numbers of positive animals given in the bars.

From 1197 samples (844 animal samples and 353 FSs), we obtained 144 *A. baumannii* isolates. As PFGE analysis identified 18 of these isolates as copy strains (identical PFGE pattern – a maximum of three strains per animal), further analysis was performed with 126 “unique” strains. The overall prevalence of *A. baumannii* in 422 cattle was 15.6% (CI: 95%, 12.3–19.5%). The rate of false positive *A. baumannii* identification with MALDI-TOF was 2.3% (two *A. nosocomialis*, one *A. calcoaceticus*). We identified considerable differences in the prevalence of *A. baumannii* with respect to the sample location (*p*-values of <0.0001 to 0.0010, Table 1). The highest prevalence was determined for NS (15.2%), followed by FSs (FS: 9.2%) and RSs (RS 1.2%). The occurrence in each sample location was dependent on the occurrence in all other sample locations (*p*-values of <0.0001 to 0.0064, Table 1). The frequency of *A. baumannii* was highest in the category “dairy cows” followed by “beef cattle” and “calves” (21.1, 6.8, and 2.4%, *p*-value: <0.0001). The three animal categories also differed in further aspects, e.g., the systemic use of 3rd generation cephalosporins in a 6 months period prior to sampling on the farm (23% in dairy cattle, 4% in beef cattle, and 21% in calves) and naturally, the age of animals (means of 58 months in dairy cattle, 19 months in beef cattle and 2 months in calves). The prevalence also differed depending on the season of sampling but not for the different geographical regions. The prevalence was highest in the 2nd trimester of the year (May–August, 44.3%), followed by the 3rd trimester (September–December, 15.3%) and the 1st trimester (January–April, 5.1%, Figure 1). At the farm level, 45 of 353 farms were positive for *A. baumannii* in the FS (12.8%), again with the highest prevalence in the 2nd trimester (26.2%) followed by the 3rd and the 1st trimester (12.3 and 2.5%, respectively).

**Table 1A:** Difference and dependency of the prevalence of *Acinetobacter baumannii* in nose swabs vs. rectal swabs.

	Negative NS	Positive NS	Sum
Negative RS	357 (84.6%)	60 (14.2%)	417 (98.8%)
Positive RS	1 (0.2%)	4 (0.9%)	5 (1.2%)
Sum	358 (84.8%)	64 (15.2%)	422 (100%)

*χ^2^-squared = 11.8252, df = 1, p-value = 0.00058. Fisher’s Exact Test for Count Data : p-value = 0.00216. McNemar’s chi-squared = 55.1475, df = 1, p-value = 0.0001. NS, nasal swabs (n = 422); RS, rectal swabs (n = 422); FS, Pen-floor fecal sample (n = 353). Only one FS was taken per farm so in some cases several NS and RS confer to the same FS.*

**Table 1B:** Difference and dependency of the prevalence of *A. baumannii* in nose swabs vs. pen-floor fecal samples.

	Negative NS	Positive NS	Sum
Negative FS	341 (80.8%)	42 (10%)	383 (90.8%)
Positive FS	17 (4%)	22 (5.2%)	39 (9.2%)
Sum	358 (84.8%)	64 (15.2%)	422 (100%)

*χ^2^-squared = 53.3386, df = 1, p-value = 0.0001. McNemar’s chi-squared = 9.7627, df = 1, p-value = 0.00178. NS, nasal swabs (n = 422); RS, rectal swabs (n = 422); FS, pen-floor fecal sample (n = 353). Only one FS was taken per farm so in some cases several NS and RS confer to the same FS.*

**Table 1C:** Difference and dependency of the prevalence of *A. baumannii* in rectal swabs vs. pen-floor fecal samples.

	Negative RS	Positive RS	Sum
Negative FS	381 (90.3%)	2 (0.5%)	383 (90.8%)
Positive FS	36 (8.5%)	3 (0.7%)	39 (9.2%)
Sum	417 (98.8%)	5 (1.2%)	422 (100%)

*χ^2^-squared = 10.0217, df = 1, p-value = 0.00155. Fisher’s Exact Test for Count Data: p-value = 0.00643. McNemar’s chi-squared = 28.6579, df = 1, p-value = 0.0001. NS, nasal swabs (n = 422); RS, rectal swabs (n = 422); FS, pen-floor fecal sample (n = 353). Only one FS was taken per farm so in some cases several NS and RS confer to the same FS.*

### Analysis of Putative Determining Factors

#### Prevalence in Cattle (All Categories Included)

The single-factor logistic regression analysis revealed nine factors that may have an influence on the occurrence of A. baumannii in the animals (Table 2). However, for only three of these factors significant values remained in the multiple logistic regression model (*n* = 387), namely systemic use of 3rd generation cephalosporins in a 6 months period prior to sampling on farm level (*p*-value: 0.0069, OR = 2.6), sampling trimester (May–August: *p*-value: <0.0001, OR = 18; September–December: *p*-value: 0.0168, OR = 2.9), and the category (calf: *p*-value: 0.0012, OR = 0.08).

**Table 2:** Results of the single-factor logistic regression for the occurrence of *A. baumannii*.

	All categories		Dairy cows		Pen-floor fecal samples	
Putative determining factor	*p*-value	nObs	*p*-value	nObs	*p*-value	nObs
Age of the animal	0.0002	422	n.s.			
Category (dairy/beef/calf)	0.0003	422				
Feeding of corn silage	0.00705	367	0.0270	266		
Feeding of straw	0.0215	105	n.s.			
Local use of 1st CEPH on farm level	n.s.		n.s.		0.0458	330
Sex of the animal	0.0028	422	n.s.			
Systemic use of 3rd CEPH on farm level	0.0010	387	0.0040	255	0.0372	319
Systemic use of florfenicol on farm level	n.s.		0.0500	255	n.s.	
Systemic use of penicillin on farm level	0.0369	387	n.s.		n.s.	
Trimester	<0.0001	422	<0.0001	280	0.0004	353
Use of sewage sludge	<0.0001	263	0.0013	169	0.0173	210

*nObs, number of observations; 3rd CEPH, 3rd generation cephalosporins; 1st CEPH, 1st generation cephalosporins; n.s., not significant.*

#### Occurrence in Dairy Cows

In dairy cows, the single-factor logistic regression analysis revealed five putative determining factors (Table 2). Out of these factors the season of sampling (May–August: *p*-value < 0.0001, OR = 22; September–December: *p*-value: 0.0130, OR = 3.7) and the use of 3rd generation cephalosporins in a 6 months period prior to sampling (*p*-value: 0.0071, OR = 2.8) were consistently significant in the multiple model (*n* = 245).

#### Occurrence in Pen-Floor Fecal Sample

The single-factor logistic regression analysis revealed four factors that may have an influence on the occurrence of *A. baumannii* in the FSs (Table 2). In the multiple model (*n* = 306) only the trimester of sampling was significant (2nd Trimester May–August; *p*-value: 0.0013; OR = 7.2).

### Antimicrobial Susceptibility and Resistance Genes

All 126 *A. baumannii* isolates showed resistance against ampicillin, amoxicillin-clavulanic acid, cefalexin, ceftiofur, nitrofurantoin and chloramphenicol. Intermediate resistance was determined for piperacillin (6%) and rifampicin (25%). All isolates were susceptible to aminoglycosides, fluoroquinolones, polymyxin B and carbapenems (Supplementary Table S5). According to PCR analysis, neither an acquired carbapenemase gene nor insertion sequences upstream of *bla*_OXA-51_ were present among the isolates.

### Phylogenetic Analysis

**FIGURE 2:**
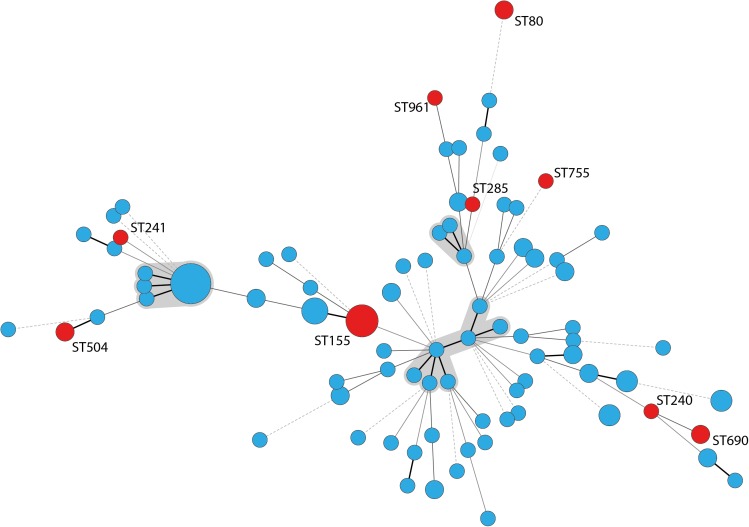
Minimum spanning tree of 126 *A. baumannii* cattle field isolates. STs that have been previously found in human samples are labeled with the ST number and circles indicating the STs are marked in red; clonal complexes are shaded gray; thick lines: single locus variant, thin lines: double locus variant, dashed line: multiple locus variants.

According to PCR analyses, 19 *A. baumannii* isolates (15%) belonged to IC2 and 16 isolates (13%) to IC3.The remaining 91 isolates were non-IC1-3 strains. The isolates did not cluster in any clonal complex (CC) that has previously been associated with IC1 to IC8 according to MLST analysis. We identified 83 different STs whereof 67 were newly described, like the most frequently identified ST1027 which forms a new CC together with its single locus variants (SLVs) ST1026, ST1033, and ST1070 (all newly described). Nine STs, including ST155, ST80, ST504, and ST690 have been previously described for human clinical *A. baumannii* strains and these STs are indicated in red in Figure 2. According to eBurst analysis, the majority of the bovine *A. baumannii* strains did not form CCs, but mostly appeared as singletons (Supplementary Figure S1). However, some bovine STs grouped together with STs recently determined for strains isolated from human specimen. For example, IHIT31974 and IHIT32879 (ST690) that were isolated from the nose of two cattle on the same farm are SLVs of ST402, which belongs to IC7 and CC25, a clonal group that has recently been linked with hospital-associated infections in humans (Sahl et al., 2015). The disperse grouping distant from the important clinical STs 1, 2, and 25 is also demonstrated by an alignment of the concated sequences of the MLST alleles (Supplementary Figure S2).

## Discussion

### Prevalence of *A. baumannii* in Hessian Cattle

The aim of the present study was to assess the prevalence of *A. baumannii* in the German state of Hesse. Only a few studies investigated the prevalence of *A. baumannii* or other *Acinetobacter* spp. in cattle before. These are only to a limited extend comparable to our study. Either sample material was restricted to fecal samples and selective media was used for initial screening, resulting in a very low recovery rate compared to our study of 0.6% (Webb et al., 2016), or information needed to determine the prevalence of *A. baumannii* in the respective species and sample locations is not provided (Hamouda et al., 2011). One study reports a very high isolation rate of 83% in mouth swabs from cattle from the Reunion Islands (Pailhoriès et al., 2016), but it only included six animals on a single farm. Probably this is not representative for a larger population of cattle in this region. A frequency similar to our results (isolation rates up to 44.3% in the second trimester) is reported by Wilharm et al. (2017) in stork samples collected in Poland with an isolation rate of 25% and local rates up to 48%.

### Prevalence in Different Categories and Different Sample Locations

With dairy cows, beef cattle, and calves, we included three different categories of cattle which differ in relevant aspects, such as feeding, lifetime, and antimicrobial treatment, probably influencing the frequency of *A. baumannii*. The highest prevalence of *A. baumannii* was observed in dairy cows (21.1%). The longer life span of dairy cows compared to beef cattle and calves, combined with a selection pressure due to use of antimicrobials (e.g., 3rd generation cephalosporins) could lead to a higher chance for dairy cows to acquire *A. baumannii* and to establish a stable colonization in the nose or gut. We isolated *A. baumannii* mainly from the nose, which could suggest that the bacterium has a tropism to the nasal mucosa. Consistent with this, *A. baumannii* has been isolated from human nasal samples with rates from 54 to 92% in long term facilities (Liou et al., 2017). However, it has been shown in another study that the colonization of the human digestive tract was also high (41%) in a nosocomial context (Corbella et al., 1996). Few studies investigated the occurrence of the bacterium in skin, nose, and fecal swabs from healthy humans and reported recovery rates of 0–10.4% (Berlau et al., 1999; Patil and Chopade, 2001; Dijkshoorn et al., 2005; Griffith et al., 2006). The high prevalence of *A. baumannii* in the cattle nose may be due to repeated acquisition of the bacterium probably by uptake or inhaling of feed particles or surrounding air contaminated with *A. baumannii*. Indeed, airborne transmission has been shown for *A. baumannii* in hospitals (Shamsizadeh et al., 2017; Solomon et al., 2017), and contamination of air samples from animal stables with *Acinetobacter* spp. has been reported (Andersson et al., 1999; Zucker et al., 2000). Moreover, *Acinetobacter* spp. have been identified in plants, including the rhizosphere of maize and others (Gulati et al., 2010; Dematheis et al., 2012), maize silage (Li and Nishino, 2011) and soybean diet in fish (Catalán et al., 2017), all of which are used as feed in cattle production.

### Identification of Putative Determining Factors

The aim of the multiple logistic regression analysis was to identify putative determining factors that could influence the occurrence of *A. baumannii* in our study population.

#### Seasonality

The analysis suggested that the season is a significant factor for the occurrence of *A. baumannii*, probably due to prolonged survival and increased growth of the bacterium associated with higher temperature and humidity. This could also explain the high prevalence in the study from the tropical Reunion Islands mentioned above (Pailhoriès et al., 2016). The seasonality of *Acinetobacter* has furthermore been reported in publications from human medicine (Retailliau et al., 1979; Poutanen et al., 1997; Perencevich et al., 2008). According to Perencevich et al. (2008) monthly infection rates for *A. baumannii* can rise by 17% for each 5.6°C increase in temperature. Interestingly, it was demonstrated that non-MDR *A. baumannii* were identified more frequently in warm months, while MDR strains showed less seasonal variation (Fukuta et al., 2012).

#### Use of 3rd Generation Cephalosporins

The chance of isolating *A. baumannii* in our sample was also higher when 3rd generation cephalosporins were used on the farm during the 6 months prior to sampling. Due to the resistance of the bovine *A. baumannii* against many cephalosporins, including the 3rd gen. cephalosporin ceftiofur, which is widely used in dairy production (24.8% of 387 animals of the sample population), selective antibiotic pressure could be a relevant factor in the maintenance of *A. baumannii* on cattle farms.

#### Use of Sewage Sludge as Fertilizer

Another interesting determining factor was the use of sewage sludge as a fertilizer on the farm. Due to the reduced number of observations (<90% of the possible observations) it was excluded from the multiple models. Though, this variable showed significant *p*-values in all single-factor analyzes and also if included to the multiple models (data not published). Distribution of *A. baumannii* via sewage sludge on feeding plants via fertilization seems possible as it has been demonstrated that it can be emitted from hospitals into the environment via wastewaters and urban sewage (Seruga Music et al., 2017) and is able to persist through the wastewater treatment process (Hrenovic et al., 2016). Our further analysis of the bovine *A. baumannii* showed that the isolates differ from human clinical isolates concerning antimicrobial resistance and phylogeny. Therefore, further research is needed to clarify (a) the role of the use of sewage sludge fertilization as a putative determining factor for *A. baumannii* in cattle and (b) the phylogeny of human non-clinical isolates.

### Antimicrobial Susceptibility

Although 50 isolates showed growth on Mueller–Hinton agar containing 2 mg/L meropenem and 10 isolates showed growth on Mueller–Hinton agar containing 4 mg/L meropenem, we could not detect phenotypic resistance to carbapenems in our isolates, as determined by a reference method for susceptibility testing. This is in concordance with other studies investigating the occurrence of *A. baumannii* in cattle without using selective media (Hamouda et al., 2011; Rafei et al., 2015). This situation seems to distinguish the cattle isolates from highly resistant strains found in companion animals and humans in a nosocomial background (Francey, 2000; Ewers et al., 2017). Regarding their antimicrobial resistance and phylogenetic diversity, *A. baumannii* strains from cattle rather resemble environmental strains and isolates from wildlife animals (Pailhoriès et al., 2015; Bardbari et al., 2017; Wilharm et al., 2017).

### Phylogenetic Analyzes

Multilocus sequence typing analysis underlined the huge diversity of *A. baumannii* strains in the investigated cattle population. Similar results were obtained in studies from Lebanon and France (Al Atrouni et al., 2016b; Pailhoriès et al., 2016). Notably, some of our bovine strains showed phylogenetic proximity to successful clinical STs. For example, IHIT32879 (ST690: 3-3-2-1-7-2-14) is a SLV of the imipenem-susceptible human clinical isolate LUH7841 (ST402: 3-3-2-1-7-2-4), which belongs to the globally distributed clinically relevant CC25 (Sahl et al., 2015). This CC corresponds to IC7 and is frequently found in human isolates (Chagas et al., 2014; Sahl et al., 2015). Several other STs have recently been isolated from humans, including carbapenem resistant isolates. ST155 has been isolated in China, the United States, and Italy from human samples and was in one occasion associated with a PER-1 gene^4^. ST80 has been identified for the first time in 2014 in OXA-40 producing carbapenem-resistant isolates from human patients in Spain (Mosqueda et al., 2014). A study from Spain identified ST80 as an important group in a Spanish hospital besides ST2, ST3, and ST15 (Villalón et al., 2015). The association to OXA-40 has also been shown for ST285 and ST755, isolated from the United States, Lebanon, and Vietnam (Table 3 and Figure 2). IHIT31924 (ST1074) is a SLV of ST135 which is part of the CC499. The other members of CC499 have already been found in human specimens and OXA-23 has been identified in one of its members (ST192). Cattle might thus host carbapenem-susceptible progenitors of clinically relevant human *A. baumannii* strains and putative transmission routes should be part of future investigations. On the other hand, the lack of the dominant human lineages such as ST2 among our bovine field isolates suggests a selection of these strains inside the hospitals rather than a transition from the cattle population. This represents a notable difference to the *A. baumannii* population in hospitalized companion animals. These animals often show the same STs (or at least ICs) and resistance mechanisms as human nosocomial isolates, indicating interspecies transmission (Endimiani et al., 2011; Zordan, 2011; Pomba et al., 2014; Ewers et al., 2016, 2017).

**Table 3:** Sequence types of bovine *A. baumannii* isolates with known occurrence in human isolates.

STPast	Number of cattle isolates	Human sample location	Acquired β-lactamase	Year of isolation	Country	Reference
ST80	2	Unknown	OXA-40	1999–2010	Spain	Villalón et al., 2011; Mosqueda et al., 2014
ST155	8	Wound	PER-1	2002, 2009, 2012	United States, China, Italy	http://pubmlst.org/abaumannii/
ST240	1	Unknown	Unknown	2010, 2013	Japan	Higuchi et al., 2014
ST241	1	Blood, sputum, stool	Unknown	Unknown	United States, Brazil	http://pubmlst.org/abaumannii/
ST285	1	Urine, sputum	OXA-40	2010	United States, Lebanon	Rafei et al., 2014
ST504	2	Perirectal	Unknown	Unknown	United States	Gregorio et al., 2015
ST690	2	Wound	Unknown	2002, 2014	Spain, Lebanon	http://pubmlst.org/abaumannii/; Al Atrouni et al., 2016a
ST755	1	n.n.	OXA-40	2009–2012	Vietnam	Schultz et al., 2016
ST961	1	Wound	Unknown	2006	United States	http://pubmlst.org/abaumannii/

## Conclusion

Our data show that *A. baumannii* is a frequent *Acinetobacter* species in cattle. In contrast to human and small veterinary medicine where primarily carbapenem-resistant isolates belonging to IC1, IC2, and IC7 cause epidemic and endemic outbreaks, the population of bovine *A. baumannii* is highly diverse and still susceptible to many antibiotics which is similar to those found in avian sources (Wilharm et al., 2017). Nevertheless, a minority of strains is phylogenetically connected to clinical isolates, but still lack acquired carbapenemase genes. These strains are a potential threat for public health especially if they enter the clinical environment and acquire resistance genes. Our data strongly suggest that the seasonal occurrence of *A. baumannii* should be taken into account in future study designs. A more detailed, genome-based characterization of bovine isolates in the context of *A. baumannii* strains isolated from humans would be of utmost importance for public health issues. It will help to understand the evolution of *A. baumannii* and might contribute to the identification of factors responsible for the assumed shift from environmental strains toward nosocomial lineages. It remains unclear where the bovine strains originate from and if they colonize the animals transiently or for a longer period. The high prevalence of *A. baumannii* in cattle nose samples, the diversity of strains isolated from individual animals, and their seasonal isolation peaks, point toward a temporarily colonization and a frequent exchange with the environment. The analysis of the meta-data hint toward uptake via the feed possibly enhanced through the fertilization with sewage sludge. This possibly forms a continuous circle of reinfection inside the cattle population.

### Limitations

Our study design comprises limitations concerning technical and statistical aspects: for productivity reasons only one colony among similar morphologies was chosen for further analysis and selective media for carbapenem-resistant strains were not included. Thus, we accepted possible underestimation of diversity and phenotypic detection of carbapenem resistant strains. Metadata were collected via questionnaires which are inevitable reliant on subjective assessments of the farmers. This resulted in missing values and mandatory exclusion of some variables from the single-factor logistic regression to the multiple models. As we did not distinguish between calves from dairy cows and beef cattle the impact of different management variables in these categories cannot be tested in our model but are met to some extend in our metadata analysis without a significant impact on the prevalence of *A. baumannii*.

## Ethics Statement

This study was carried out in accordance with the recommendations of the directive 2010/63/EU of the European parliament and of the council and the German animal welfare law. According to the assessment of the animal welfare officer of the Faculty of Veterinary Medicine of Giessen in 2014 the animals did not experience pain, suffering, distress, or lasting harm due to the sampling. An ethical committee statement was therefore not necessary.

## Author Contributions

PK, KF, SS, TS, and CE contributed conception and design of the study. PK collected the samples, organized the database, and wrote the first draft of the manuscript. KF wrote sections of the manuscript. PK, KF, and AS performed the statistical analyzes. PK, PH, and HS performed the MLST analyzes. PK and UL performed the PFGE and PCR experiments. All authors contributed to manuscript revision, read and approved the submitted version.

## Conflict of Interest Statement

The authors declare that the research was conducted in the absence of any commercial or financial relationships that could be construed as a potential conflict of interest.

## Footnotes

1 www.hi-tier.de

2 http://www.r-project.org

3 http://pubmlst.org

4 https://pubmlst.org/abaumannii/

## Abbreviations

FS: pen-floor fecal sample
NS: nasal swab
RS: rectal swab.
