# Supplementary figures and images for: Seasonal Occurrence and Carbapenem Susceptibility of Bovine Acinetobacter baumannii in Germany
Source: Front Microbiol. 2019 Feb 22;10:272. doi: 10.3389/fmicb.2019.00272 (PMC6395434; doi:10.3389/fmicb.2019.00272)

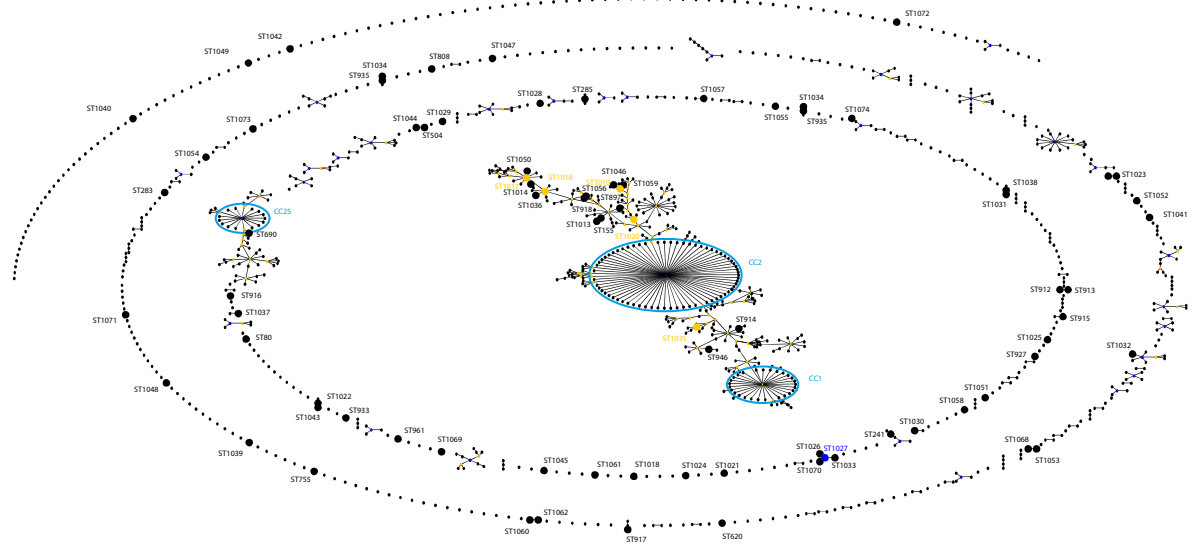

Supplement: Supplementary file 1 [file Data_Sheet_1.PDF]

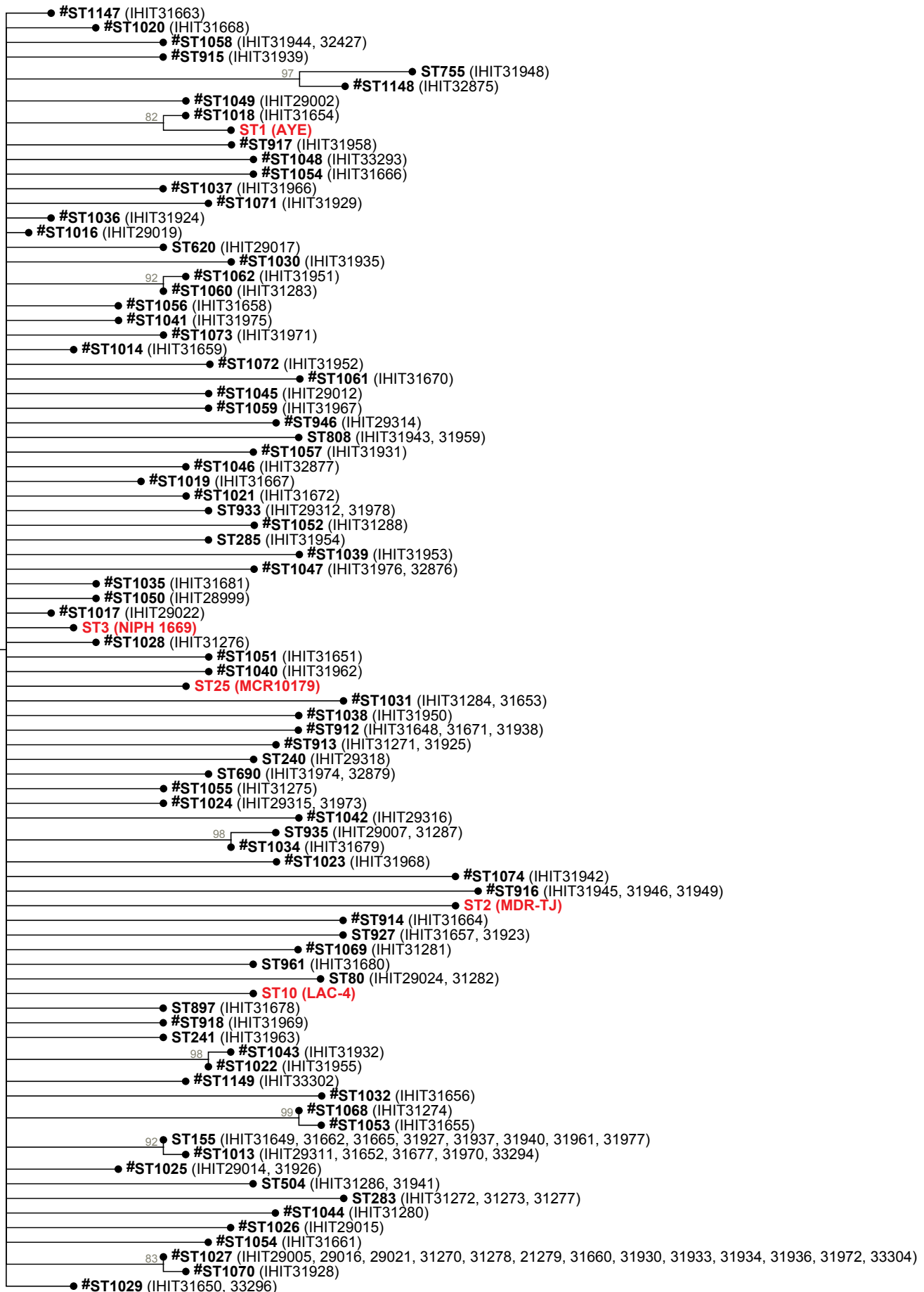

0.001

Supplement: Supplementary file 2 [file Data_Sheet_2.PDF]
