# Supplementary material for: Seasonal Occurrence and Carbapenem Susceptibility of Bovine Acinetobacter baumannii in Germany
Source: Front Microbiol. 2019 Feb 22;10:272. doi: 10.3389/fmicb.2019.00272 (PMC6395434; doi:10.3389/fmicb.2019.00272)
Supplement: Supplementary file 3 [file Table_1.doc]

**Supplemental material:**

**Table S1. Numbers of animals according to the study design showing the stratification**

|  | **Number of animals** | | | | | | |
| --- | --- | --- | --- | --- | --- | --- | --- |
|  | **Total** | **”Dairy”** | | **”beef”** | | **”Calf”** | |
| **District** | **pop.** | **pop.** | **sample** | **pop.** | **sample** | **pop.** | **sample** |
| Darmstadt | 928 | 569 | 1 | 187 | 0 | 172 | 0 |
| Bergstrasse | 1,1029 | 6,408 | 6 | 2,116 | 2 | 2,505 | 2 |
| Darmstadt Dieburg | 5,920 | 2,495 | 2 | 2,104 | 3 | 1,321 | 2 |
| Gross-Gerau | 1,319 | 550 | 1 | 502 | 0 | 267 | 0 |
| Hochtaunuskreis | 2,551 | 1,601 | 2 | 414 | 0 | 536 | 1 |
| Main-Kinzig-Kreis | 26,105 | 15,706 | 16 | 5,004 | 6 | 5,395 | 5 |
| Main-Taunus-Kreis | 863 | 458 | 0 | 213 | 0 | 192 | 1 |
| Odenwaldkreis | 17,481 | 11,484 | 11 | 2,393 | 2 | 3,604 | 4 |
| Offenbach | 2,016 | 1,396 | 1 | 299 | 0 | 321 | 0 |
| Rheingau-Taunus-Kreis | 1,515 | 681 | 1 | 376 | 0 | 458 | 0 |
| Wetteraukreis | 15,616 | 9,498 | 9 | 2,901 | 3 | 3,217 | 3 |
| Giessen | 10,292 | 6,911 | 7 | 1,356 | 1 | 2,025 | 2 |
| Lahn-Dill-Kreis | 8,055 | 4,510 | 4 | 1,242 | 1 | 2,303 | 2 |
| Limburg-Weilburg | 14,253 | 9,439 | 9 | 2,154 | 2 | 2,660 | 3 |
| Marburg-Biedenkopf | 24,695 | 15,757 | 16 | 4,106 | 4 | 4,832 | 5 |
| Vogelsbergkreis | 47,102 | 32,592 | 32 | 5,913 | 6 | 8,597 | 9 |
| Fulda | 59,346 | 39,743 | 39 | 7,411 | 7 | 12,192 | 12 |
| Hersfeld-Rotenburg | 18,349 | 11,203 | 11 | 3,279 | 3 | 3,867 | 4 |
| Kassel | 16,279 | 10,937 | 11 | 2,442 | 2 | 2,900 | 3 |
| Schwalm-Eder-Kreis | 28,514 | 20,112 | 20 | 3,178 | 3 | 5,224 | 5 |
| Waldeck-Frankenberg | 55,501 | 41,173 | 41 | 4,636 | 5 | 9,692 | 10 |
| Werra-Meissner-Kreis | 16,141 | 11,622 | 12 | 1,624 | 2 | 2,895 | 3 |
| **Total** | **383,870** | **254,845** | **252** | **52,226** | **50** | **72,280** | **73** |

**pop. = population**

**Table S2: Questionnaire for collection of metadata**

| **Questionnaire sampling of cattle**  **Farm: _____________________** | | **Number:** |
| --- | --- | --- |
| **Individual Number:** | **Race:**   - **HF** - **FV** - **__________** | **Sex:**   - **Male** - **Female** |
| **Age:** | **Category:**   - **Dairy** - **Feedlot** - **Calf** | **Pre-existing illness:**   - **None** - **__________** |
| **Pre-treatment:**   - **None** - **__________** | **Use of antibiograms:** |  |
| **Keeping:**   - **Tethering** - **Pen** - **Pasture** - **_____________** | **Size of farm:**  **Animals in total:_____**  **Cows: _____________**  **Young cattle:_______**  **Feedlot: ___________** | **Field of use:**   - **Dairy farm** - **Fattening farm** - **Suckler cow husbandry** - **______________** |
| **Organic Farming:**   - **conventional** - **“Demeter”** - **“Bioland”** - **“Naturland”** - **_________** | **Feeding:**   - **Wastemilk (Calves)** - **Silage (gras/ corn)** - **Rap/Soy** - **____________** - **____________** | **Fertilizer:**   - **Sewage sludge** - **Biogas plant slurry** - **neither** |
| **Application of antibiotic drying agents:**   - **Every cow** - **Regularly** - **Seldom** - **Never** | **Locally administred antibiotics used on farm in the last 6 months:**  **______________________**  **______________________**  **______________________**  **______________________** | **Systemically administred antibiotics used on farm in the last 6 months:**  **______________________**  **______________________**  **______________________**  **______________________** |
| **Subjective assessment of the administration of antibiotics:**   - **Once a week** - **Once a month** - **Once half a year** - **Once a year** | **Contact to other animals:**   - **Dogs** - **Cats** - **Birds** - **wild animals** | **Pre-treatment of associated animals:**   - **None** - **_______________** |
| **Last hospital visit of the owner:**   - **None in the past half a year** - **during the last month** - **during the last 3 months** - **during the last 6 months** | **Intake of antibiotics - owner:**   - **Less than once a year** - **Once a week** - **Once a month** - **Once half a year** - **Once a year** | **Antibiotics used by the owner int the las 6 months:**  **______________________**  **______________________**  **______________________**  **______________________** |
| **I agree with the sampling and the pseudonymized use of the data for the dissertation of Mr. Peter Klotz. Furthermore, I am aware of the fact, that the participation is voluntary and can be retracted anytime.**  **__________________**  **Signature** | | |

**Table S3: Putative determining factors included in logistic regression model**

| **#** | **Variable Name** | **Description** |
| --- | --- | --- |
| 1 | Monat | Month of sampling |
| 2 | PLZ | ZIP-Code |
| 3 | LDKR | Rural district |
| 4 | RegBZ | Govermental district |
| 5 | Rasse | Breed |
| 6 | Sex | Sex |
| 7 | AlterKat | Age categorized |
| 8 | Alter | Age in months |
| 9 | Kategorie | Category "dairy", "beef", "calf" |
| 10 | Deck | Breeding bull yes/no |
| 11 | Vorkrank_ja_nein | Diseases diagnosed in animal prior to sampling |
| 12 | Vorerkrank_Kat | Categorisation of prior diseases |
| 13 | Vorbehand1 | Treatment prior to sampling |
| 14 | Vorbehand_Kat | Categorisation of pre-treatment |
| 15 | Penicillin | Treatment with penicillin |
| 16 | Cephalosporin1 | Treatment with cephalosporin 1^st^ generation |
| 17 | Cephalosporin3 | Treatment with cephalosporin 3^rd^ generation |
| 18 | Cephalosporin4 | Treatment with cephalosporin 4^th^ generation |
| 19 | Aminoglycoside | Treatment with aminoglycosides |
| 20 | Tetracycline | Treatment with tetracyclines |
| 21 | Sulfonamide | Treatment with sulfonamides |
| 22 | Makrolide | Treatment with macrolides |
| 23 | Fluorchinolone | Treatment with fluoroquinolones |
| 24 | Polymyxine | Treatment with polymyxins |
| 25 | Florfenicol | Treatment with florfenicole |
| 26 | Antibio1 | Use of antibiograms prior to antibiotic treatment |
| 27 | Haltung1 | Husbandry system |
| 28 | Haltung_Boden | Type of floor in husbandry |
| 29 | Haltung_Box | Type of box in husbandry |
| 30 | BetriebsgrKat | Size off arm categorized |
| 31 | BetriebsgrGes | Number of animals in total |
| 32 | BetriebsgrK | Number of dairy cows |
| 33 | BetriebsgrJ | Number of young cattle |
| 34 | BetriebsgrM | Number of beef cattle |
| 35 | Nutzung | Direction of use oft he farm |
| 36 | Bio | Type of ecological guidelines |
| 37 | Bio_binär | Application of ecological guidelines indepent of type |
| 38 | Gras | Feeding of pasture |
| 39 | Grassilage | Feeding of ensilaged pasture |
| 40 | Maissilage | Feeding of ensilaged corn |
| 41 | Heu | Feeding of hay |
| 42 | Stroh | Feeding of straw |
| 43 | Raps | Feeding of rapeseed |
| 44 | Soja | Feeding of soy |
| 45 | Biertreber | Feeding of draff |
| 46 | KFzukauf | Feeding of commercial concentrate |
| 47 | KFeigen | Feeding of selfmade concentrate |
| 48 | Mineralfutter | Feeding of mineral feed |
| 49 | Käberfutter1 | Feeding of calves with milkpowder or whole milk |
| 50 | Sperrmilch | Feeding of waste milk |
| 51 | Düngung | Fertilization with either sewage sludge, biogags plant slurry, both, or neither |
| 52 | TS1_Bestand | Use of antibiotic drying agents |
| 53 | TS_Kat_Bestand | Antibiotic class of used drying agent |
| 54 | Versiegler | Use of intramammary seal product |
| 55 | LokAntibio1 | Use of local antibiotics in the last 6 months on the farm |
| 56 | Penicillin_Lok | Local use of penicillins on the farm |
| 57 | Cephalosporin1_Lok | Local use of 1^st^ generation cephalosporins on the farm |
| 58 | Cephalosporin3_Lok | Local use of 3^rd^ generation cephalosporins on the farm |
| 59 | Cephalosporin4_Lok | Local use of 4^th^ generation cephalosporins on the farm |
| 60 | Aminoglykoside_Lok | Local use of aminoglycosides on the farm |
| 61 | Lincosamide_Lok | Local use of lincosamydes on the farm |
| 62 | Tetracycline_Lok | Local use of tetracyclines on the farm |
| 63 | Sulfonamide_Lok | Local use of sulfonamides on the farm |
| 64 | Fluorchinolone_Lok | Local use of fluoroquinolones on the farm |
| 65 | Florfenciol_Lok | Local use of florfenicole on the farm |
| 66 | SysAntibio1 | Use of systemic antibiotics in the last 6 months on the farm |
| 67 | Penicillin_Sys | Systemic use of penicillins on the farm |
| 68 | Cephalosporine1_Sys | Systemic use of 1^st^ generation cephalosporins on the farm |
| 69 | Cephalosporine3_Sys | Systemic use of 3^rd^ generation cephalosporins on the farm |
| 70 | Cephalosporine4_Sys | Systemic use of 4^th^ generation cephalosporins on the farm |
| 71 | Aminoglykoside_Sys | Systemic use of aminoglycosides on the farm |
| 72 | Tetracycline_Sys | Systemic use of tetracyclines on the farm |
| 73 | Sulfonamide_Sys | Systemic use of sulfonamides on the farm |
| 74 | Makrolide_Sys | Systemic use of macrolides on the farm |
| 75 | Fluorchinolone_Sys | Systemic use of fluoroquinolones on the farm |
| 76 | Polymyxine_Sys | Systemic use of polymyxins on the farm |
| 77 | Florfenicol_Sys | Systemic use of florfenicoles on the farm |
| 78 | AntibioFreq1 | Frequency of use of antibiotics (subjective assessment) |
| 79 | Tierkont | Contact of cattle to other animals |
| 80 | VorbehKont1 | Pre-treatment of contact animals |
| 81 | KHalter1 | Last hospitalization of the farmer |
| 82 | AntbioFrequH | Frequency of personal use of antibiotics (subjective assessment) |
| 83 | AntbioH1 | Personal use of antibiotics in the last six months |

**Table S4: Number of animals sampled, showing the stratification of the sample Number of animals in sample**

| **District** | **Number of animals in sample** | | |
| --- | --- | --- | --- |
|  | **“dairy”** | **“beef”** | **“calf”** |
| Darmstadt | 0 | 0 | 0 |
| Bergstraße | 6 | 2 | 3 |
| Darmstadt Dieburg | 3 | 3 | 2 |
| Groß-Gerau | 1 | 0 | 0 |
| Hochtaunuskreis | 2 | 1 | 1 |
| Main-Kinzig-kreis | 18 | 6 | 6 |
| Main-Taunus-Kreis | 0 | 0 | 1 |
| Odenwaldkreis | 13 | 3 | 4 |
| Offenbach | 1 | 0 | 0 |
| Rheingau-Taunus-Kreis | 1 | 0 | 0 |
| Wetteraukreis | 9 | 3 | 3 |
| Wiesbaden | 1 | 0 | 0 |
| Gießen | 7 | 1 | 2 |
| Lahn-Dill-Kreis | 5 | 1 | 2 |
| Limburg-Weilburg | 9 | 2 | 3 |
| Marburg-Biedenkopf | 15 | 3 | 7 |
| Vogelsbergkreis | 35 | 6 | 9 |
| Fulda | 44 | 9 | 12 |
| Hersfeld-Rotenburg | 14 | 2 | 4 |
| Kassel | 12 | 4 | 3 |
| Schwalm-Eder-Kreis | 23 | 3 | 9 |
| Waldeck-Frankenberg | 45 | 7 | 10 |
| Werra-Meißner-Kreis | 16 | 3 | 2 |
| Total | 280 | 59 | 83 |

**Numbers differ from table S1 due to variable sample conditions encountered on different farms**

**Table S5: MIC values of the *A. baumannii* isolates**

see excel file

**Figure S1**: eBurst snapshot of the all sequence types in the PubMLST Database. Dots of STs found in cattle are enlarged, **blue**: possible group founder, **yellow**: possible subgroup founder, **blue circles**: clonal complexes

**Figure S2**: Maximum-likelihood tree created with RAxML and 100 bootstrap analyses based on the concatenated sequences of seven housekeeping genes according to the MLST-Pasteur scheme of 126 bovine *A. baumannii* isolates and representatives of global sequence types ST1, ST2, ST3, ST10, and ST25 (Accession numbers: AYE- GenBank: CU459141.1, NIPH 1669: APOQ00000000.1, MDR-TJ: CP003500.1, LAC-4: JICJ00000000.1, MCR10179: NQXG01). Internal nodes with less than 80% bootstrap support were removed. Novel STs are indicated (#). Reference STs are highlighted in red.
